# A Mouse Model for Infection with Enterovirus A71 in Small Extracellular Vesicles

**DOI:** 10.1128/mSphere.00377-20

**Published:** 2020-07-01

**Authors:** Jiaqi Gu, Jing Wu, Yuwen Cao, Xinran Zou, Xiaonan Jia, Yiqian Yin, Li Shen, Daihua Fang, Lingxiang Mao

**Affiliations:** a Department of Laboratory Medicine, The Affiliated People’s Hospital, Jiangsu University, Zhenjiang, China; b Department of Immunology, Jiangsu Key Laboratory of Laboratory Medicine, School of Medicine, Jiangsu University, Zhenjiang, China; c Zhenjiang Center for Disease Control and Prevention, Zhenjiang, Jiangsu, China; d Clinical Laboratory, Xuzhou Children’s Hospital, Xuzhou, China; University of Zurich

**Keywords:** enterovirus A71 (EV-A71), mouse model, small extracellular vesicles (sEVs), central nervous system (CNS), pathogenesis

## Abstract

Enterovirus A71 (EV-A71) is the major pathogen of hand, foot, and mouth disease (HFMD); in some severe cases, it could develop into central nervous system (CNS) disease such as aseptic meningitis, encephalitis, and neurogenic pulmonary edema in children under 5 years. The EV-A71 pathogenesis which is involved with the CNS is unclear due to the lack of a simple and reliable mouse model thus far. Most clinical EV-A71 isolates could not effectively infect the neonatal mouse, which used to be an EV-A71 infection model. The small extracellular vesicles (sEVs) released from clinical EV-A71 isolate-infected cells were infectious in cell lines and could cause a high viral replication in mice. Neonatal ICR mice were injected intraperitoneally with these infectious sEVs and showed more weight loss and higher mortality than those mice injected with the clinical EV-A71 isolate. By using these sEVs, we provided a simple and effective method by which we can generate a stable and valuable animal model for the studies of EV-A71 pathogenesis and therapy.

**IMPORTANCE** EV-A71 was supposed to infect the CNS through the neural pathway and the circulation of the blood in previous studies. Reverse axon transport had been confirmed as an important pathway for EV-A71 to infect the CNS; however, it is still unknown how EV-A71 infects the CNS through the circulation of the blood. Combined with the infectivity of sEVs secreted from EV-A71-infected cells and the characteristic that sEVs could cross the blood-brain barrier, we considered that sEVs may play a vital role in EV-A71 pathogenesis of the CNS.

## Abstract (precis)

EV-A71 was supposed to infect the CNS through the neural pathway and the circulation of the blood in previous studies. Reverse axon transport had been confirmed as an important pathway for EV-A71 to infect the CNS; however, it is still unknown how EV-A71 infects the CNS through the circulation of the blood. Combined with the infectivity of sEVs secreted from EV-A71-infected cells and the characteristic that sEVs could cross the blood-brain barrier, we considered that sEVs may play a vital role in EV-A71 pathogenesis of the CNS.

## OBSERVATION

Enterovirus A71 (EV-A71), a single positive-strand RNA virus, is a member of the *Picornavirus* family and *Enterovirus* genus. This virus is the main pathogen for hand, foot, and mouth disease, which is associated with severe neurological manifestations, and it has many times been associated with outbreaks and epidemics over the world (1). However, the pathogenic mechanism of EV-A71-caused severe diseases is poorly defined due to the lack of simple and proper animal models. It is very difficult to use nonhuman primates as animal models because of the ethical and cost problem. Previous studies established infection models by mouse-adapted viruses which were generated after serial passages of the parental EV-A71 strain in mice or in mouse embryonic fibroblast NIH/3T3 cells and increased the virulence of the EV-A71 clinical isolates in mice (2, 3). However, the generation of mouse-adapted viruses is elaborate and could increase artificial mutations. Alternatively, clinical isolates would not need adaptation to transgenic mice or immunodeficient mice. However, *in vitro* infection with these special mice is limited to study the pathogenesis of EV-A71 infection in some respects.

Small extracellular vesicles (sEVs) are <200-nm particles which are released from the cells and delimited by a lipid bilayer. Exosomes are just parts of sEVs which predominantly originate from the endosomal system and have characteristic tetraspanin membrane markers (CD9, CD63, CD81).

The sEVs are now considered new extracellular functional carriers which could transport protein, nucleic acids, and lipids, etc., to neighboring cells. They also play an important role in viral spread (4, 5). Viruses could transmit the infection and evade the immune system by EVs cloaking viral proteins and viral genomes (6), such as poliovirus (7), hepatitis A virus (8), and coxsackievirus B (9), and so on. The sEV-mediated extracellular communication may play an important role in viral pathogenesis and control of host immune response to infection (10).

In consideration that sEVs could transfer their cargo across the blood-brain barrier (11), it may be related to EV-A71 infection in the CNS. We separated the sEVs from EV-A71 clinical isolate-infected cells by using differential ultracentrifugation with further isopycnic gradient centrifugation and identified the sEVs by transmission electron microscopy (TEM), nanoparticle tracking analysis (NTA), and Western blotting (WB). These sEVs containing viral RNA are infectious *in vitro* and capable of infecting neonatal immunocompetent mice. Compared with the EV-A71 clinical isolate, these infectious sEVs showed greater neurovirulence and more fatality in the mouse model with intraperitoneal injection. This present model could be generated easily by isolated sEVs and is useful for studying the pathogenesis of enterovirus-associated diseases.

### Generation of sEVs from EV-A71-infected cells.

**FIG 1:**
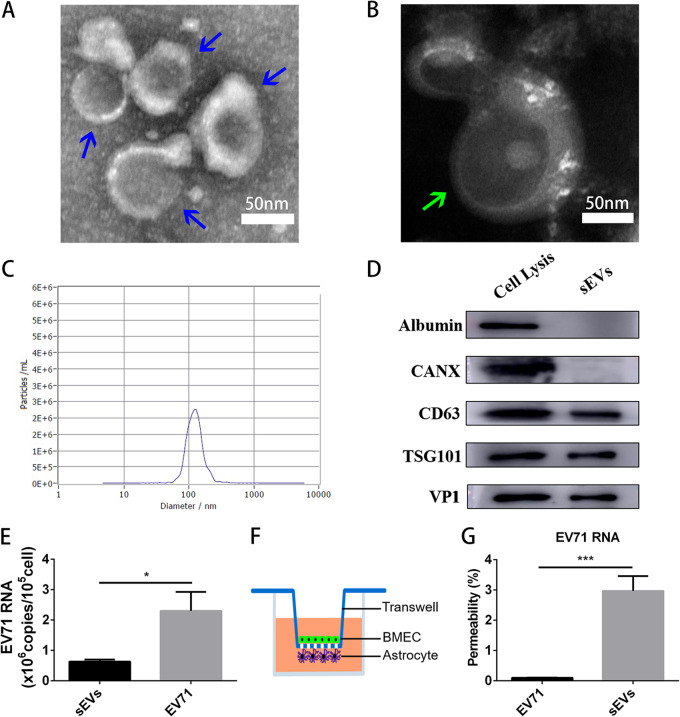
Characterization of sEVs from EV-A71-infected cells. (A and B) TEM images of empty sEVs (blue arrows) (A) and sEVs with virus-like particles (green arrow) (B). (C) Diameter of sEVs measured by NTA. (D) Immunoblotting of EV-positive markers (CD63 and TSG101), negative markers (albumin and calnexin), and viral capsid proteins (VP1) in sEVs and cell lysis. (E) Detection of viral RNA in EV-A71 and sEVs by qRT-PCR (mean ± SD; 3 independent experiments). (F) Schematic drawing of *in vitro* BBB model. (G) Comparison of permeabilities between sEVs and EV-A71 (mean ± SD; 3 independent experiments). In all panels: ns, not significant; *, *P* ≤ 0.05; **, *P* ≤ 0.01; ***, *P* ≤ 0.001; ****, *P* ≤ 0.0001.

In order to separate sEVs with high specificity, we used differential ultracentrifugation with further isopycnic gradient centrifugation to isolate sEVs from the cells infected by the clinical EV-A71 isolate. The sEVs floating at 1.09 to 1.14 g cm^−3^ were collected and identified. Transmission electron microscopy (TEM) images showed mostly empty sEVs (Fig. 1A) and a few sEVs containing virus-like particles which were 27- to 30-nm compact particles (Fig. 1B). Nanoparticle tracking analysis (NTA) showed sEVs concentrated on the size of 118.1 nm (Fig. 1C). The positive markers and negative markers of EVs were verified by immunoblotting, and the viral capsid protein VP1 was in the sEVs (Fig. 1D). qRT-PCR analysis of sEVs showed that sEVs contained EV-A71 RNA (Fig. 1E).

To study the permeability of sEVs across the blood-brain barrier (BBB), we established the *in vitro* static BBB model by coculturing brain microvascular endothelial cells (BMEC) and astrocytes from neonatal mice in the transwell (Fig. 1F). To compare permeabilities between EV-A71 and sEVs, EV-A71 and sEVs with the same quantity of EV-A71 RNA were added in the BMEC of the upper side of the BBB model, respectively, and then the level of EV-A71 RNA of the bottom side was measured by qRT-PCR after 3 h. The ratio of upper/bottom viral RNA quantity was calculated as a measure of permeability. Obviously, sEVs crossed the BBB more efficiently than EV-A71 (Fig. 1G). It suggested sEVs may play an important role in CNS infection.

### Infection of neonatal mouse with the sEV derived from clinical EV-A71 isolate.

To explore whether sEVs released from EV-A71 clinical isolate-infected cells could effectively infect the neonatal mice, we respectively inoculated sEVs and the EV-A71 clinical isolate at a dose of 1 × 10^4^ PFU/mouse by intraperitoneal injection. Compared with intracranial injection, intraperitoneal injection could avoid the influence of challenge methods on the brain and simulate the pathogenesis of EV-A71 factually.

**FIG 2:**
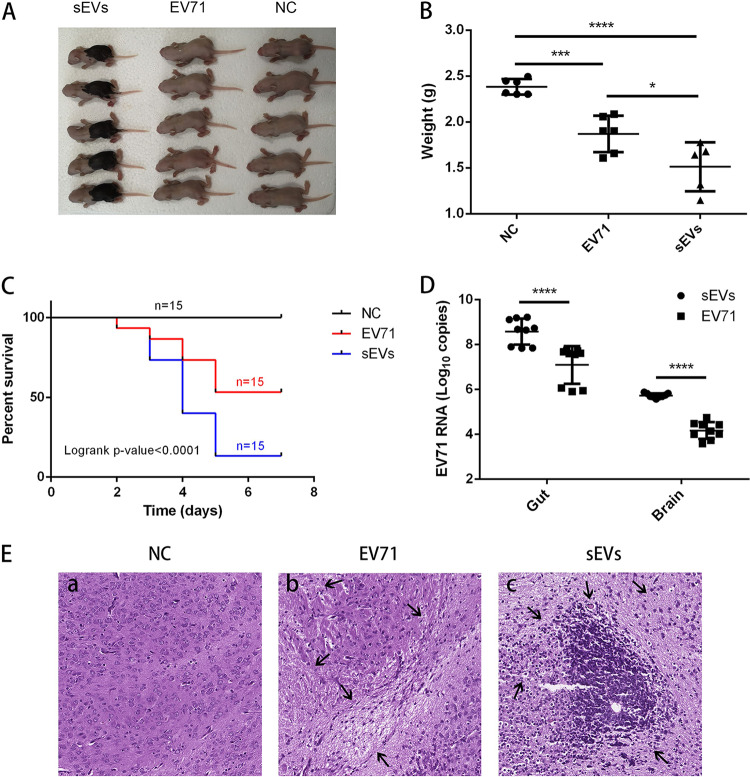
sEVs showed stronger neurovirulence than EV-A71 in the mouse model. (A and B) Size (A) and weight (B) differences of mice in different treatment groups at 3 dpi. (C) Survival curve of mice after injection. (D) Absolute quantification of EV-A71 RNA in gut and brain was performed by qRT-PCR. The virus RNA copies for various tissues of each infected mouse were shown as mean for three independent experiments. The viral RNA loads of different treatment groups were compared. Statistical analyses were performed using Mann-Whitney U test. (E) Representative H&E images of brain damage. The EV-A71-infected group exhibited mild neuropil vacuolation and neuronal loss (arrows), and the sEV-infected group exhibited numerous inflammatory cells and vesicular degeneration (arrows). A representative result is shown. In all panels: *, *P* ≤ 0.05; **, *P* ≤ 0.01; ***, *P* ≤ 0.001; ****, *P* ≤ 0.0001. NC, negative control.

At 3 days postinfection (dpi), the mice infected with sEVs lost weight obviously whereas the mice infected with EV-A71 did not lose weight apparently (Fig. 2A and B). Within 7 days, the mortality rate of mice infected with sEVs was 86.7% whereas the mortality rate of mice infected with EV-A71 was only 46.7% (Fig. 2C). To validate viral replication in tissues of infected mice, we collected the main infected tissues, the brain and gut, after the mice died by infection or euthanasia at 7 dpi and quantified the viral RNA. The viral RNA levels in brain and gut tissues in the sEV-infected group were significantly higher than those in the EV-A71-infected group (Fig. 2D). At the same time, histological examination was performed on the brain; consistent with the previous results, numerous histopathological changes were observed in the sEV-infected group: some nerve cells had fatty or vesicular degeneration and the number of inflammatory cells was increased obviously. However, the EV-A71-infected group exhibited mild neuropil vacuolation and neuronal loss in brainstem reticular formation (Fig. 2E).

These results suggested the neonatal mice were more susceptible to the sEVs than to the clinical isolate and that an effective animal model could be easily generated by the sEVs without adaptive mutations.

Since the first report that EV-A71 could infect newborn mice by intraperitoneal injection and cause paralysis, death, etc., in 1979, the EV-A71-infected mouse model has been optimized several times (12). Some studies investigated potential influence factors such as virus dose, challenge method, mouse strain, and mouse age. but the results showed that they cannot help to increase the virulence of EV-A71 (13).

The majority of studies tried to adapt EV-A71 in mice or mouse embryonic fibroblast NIH/3T3 cells because EV-A71 which was isolated from human samples failed to infect mice efficiently. The results showed this was an effective method; however, virus replication may be affected by the mouse immune system during this long process, and the disease in EV-A71-infected mice was readily attenuated by anti-EV-A71 antibody (2).

In our study, we increased EV-A71 susceptibility in mice by the sEVs released from EV-A71-infected NIH/3T3 cells which originated from mice. According to guidelines (14), sEVs were separated from the culture of EV-A71-infected cells by ultracentrifugation and isopycnic gradient centrifugation with high specificity. We inoculated EV-A71 in mice by intraperitoneal injection and monitored their clinical symptoms. The results showed sEVs could cause a higher rate of death and more severe CNS infection in neonatal mice, and the level of EV-A71 RNA replication in brain and gut of mice was higher than for EV-A71 virus. EV-A71 could only attach or/and enter the cells via binding EV-A71 receptors on the surface of the infected cells, while the sEVs could transport the cargo without any receptor. In consideration that sEVs could cross the blood-brain barrier by transcytosis (15), it is possible that the property of the infectious sEVs is helpful to infect the neonatal mice successfully.

In summary, we have developed a stable mouse model with sEVs released from EV-A71 clinical isolate-infected cells. This new model is easier to establish than before and represents a powerful tool for investigating EV-A71 neurologic pathogenesis.

### Cells and viruses.

NIH/3T3 cells were cultured in Dulbecco modified Eagle medium (DMEM) supplemented with 10% fetal bovine serum (FBS). The cell lines were obtained from ATCC. Cells were infected with EV-A71 at a multiplicity of infection (MOI) of 1 and incubated for 1 h at 37°C and 5% CO_2_. Then, cells were rinsed and further incubated with prewarmed DMEM supplemented with 5% FBS which had been ultracentrifuged to remove EVs. The cells were collected, cracked by three freeze-thawing cycles, and centrifuged at 4,000 rpm at 4°C for 10 min to remove cellular debris. Infectious virus titers were determined by 50% tissue culture infective dose (TCID_50_) assay as described elsewhere (16).

### Ethics statement.

All animal procedures were approved by the ethics committee of Jiangsu University Animal Care Committee, followed the *Guide for the Care and Use of Laboratory Animals* published by the Chinese National Institutes of Health, and were performed in compliance with the animal behavioral guidelines, using approved protocols from the institutional animal care committee.

### Establishment of BBB model.

Astrocytes were isolated from neonatal mouse, purified by cell passage, and then cocultured with BMEC. A schematic drawing of the *in vitro* BBB model is shown in Fig. 1F.

### Animal experiments.

For all experiments, 1-day-old ICR mice were purchased from the Laboratory Animal Centre of Jiangsu University. They were housed together in an environment of 50% humidity at 22°C under a 12-h light/dark cycle and kept with their mothers to provide food (17). Two groups of 1-day-old ICR mice were inoculated with EV-A71 and sEVs (1 × 10^4^ TCID_50_, 20 μl per mouse), respectively, by intraperitoneal injection. One group was administered PBS (20 μl per mice) as a control. The mice were monitored for 5 days for clinical symptoms and were sacrificed if the mice met the following criteria: lethargy, hind limb paralysis, hypothermia of <33.5°C, or death. Brain tissues were collected and fixed in 4% paraformaldehyde. After being embedded in paraffin and sliced, the brain sections (10 μm) were stained with hematoxylin and eosin (H&E) for morphological examination. Ten sections of brain were observed per mouse in a blind manner.

### qRT-PCR assay for EV-A71 RNA.

Total RNA of the cells and the tissues was extracted with TRIzol reagent (Invitrogen); RNA from culture supernatants was extracted with the RNAqueous-Micro kit (Thermo Fisher Scientific). EV-A71 was determined in a SYBR green two-step qRT-PCR assay (Bio-Rad). The primer sequencing of EV-A71 was performed as described previously (18).

### sEV separation.

Cell culture supernatant fluids were centrifuged at 1,000 × *g* at 4°C for 10 min to remove cells and debris and then centrifuged at 10,000 × *g* at 4°C for 30 min twice to remove subcellular fraction, and the sEVs were collected after ultracentrifugation at 100,000 × *g* for 1 h at 4°C. The sEVs were resuspended in PBS, loaded onto an 8 to 40% iodixanol (Opti-prep) step gradient, and centrifuged at 150,000 × *g* in an SW32TI Beckman Coulter rotor for 24 h at 4°C. One-milliliter fractions were collected, and density was determined with a refractometer (19–21).

### TEM.

Samples of 2.5 μl were adsorbed on the surface of a glow-discharged 400 mesh carbon-coated copper grid for 5 min, and the grid was fixed in 1% glutaraldehyde with 0.15 M phosphate buffer (pH 7.4) for 1 min; afterward, it was rinsed with deionized water and stained with 3% ammonium molybdate, pH 7.0.

### NTA.

The size and concentration of sEVs derived from EV-A71-infected cells were analyzed using the ZetaView PMX 110 (Particle Metrix, Meerbusch, Germany) and configured with a scientific complementary metal oxide semiconductor (CMOS) camera and blue 488-nm laser. For ZetaView analysis, sEVs were diluted in 2 ml PBS, collected, and analyzed by the ZetaView 8.04.02 SP2.

### Immunoblotting.

Cells were lysed with radioimmunoprecipitation assay (RIPA) buffer (Kangwei Century). Immunoblot assays were performed with standard procedures and the indicated antibodies. Protein bands were detected by an Image Quant LAS 4000 mini (GE Healthcare).

### Statistical analysis.

Data were analyzed by GraphPad Prism software (version 6.01). A *P* value of <0.05 was considered significant.

## References

[1] Cox JA, Hiscox JA, Solomon T, Ooi MH, Ng L. 2017. Immunopathogenesis and virus-host interactions of enterovirus 71 in patients with hand, foot and mouth disease. Front Microbiol 8:2249. doi:10.3389/fmicb.2017.02249. 29238324 PMC5713468

[2] Wang Y-F, Chou C-T, Lei H-Y, Liu C-C, Wang S-M, Yan J-J, Su I-J, Wang J-R, Yeh T-M, Chen S-H, Yu C-K. 2004. A mouse-adapted enterovirus 71 strain causes neurological disease in mice after oral infection. J Virol 78:7916–7924. doi:10.1128/JVI.78.15.7916-7924.2004. 15254164 PMC446098

[3] Chen Y-C, Yu C-K, Wang Y-F, Liu C-C, Su I-J, Lei H-Y. 2004. A murine oral enterovirus 71 infection model with central nervous system involvement. J Gen Virol 85:69–77. doi:10.1099/vir.0.19423-0. 14718621

[4] Meckes DG, Jr, Shair KHY, Marquitz AR, Kung C-P, Edwards RH, Raab-Traub N. 2010. Human tumor virus utilizes exosomes for intercellular communication. Proc Natl Acad Sci U S A 107:20370–20375. doi:10.1073/pnas.1014194107. 21059916 PMC2996715

[5] Pegtel DM, Cosmopoulos K, Thorley-Lawson DA, van Eijndhoven MAJ, Hopmans ES, Lindenberg JL, de Gruijl TD, Würdinger T, Middeldorp JM. 2010. Functional delivery of viral miRNAs via exosomes. Proc Natl Acad Sci U S A 107:6328–6333. doi:10.1073/pnas.0914843107. 20304794 PMC2851954

[6] Anderson MR, Kashanchi F, Jacobson S. 2016. Exosomes in viral disease. Neurotherapeutics 13:535–546. doi:10.1007/s13311-016-0450-6. 27324390 PMC4965413

[7] Jackson WT, Giddings TH, Jr, Taylor MP, Mulinyawe S, Rabinovitch M, Kopito RR, Kirkegaard K. 2005. Subversion of cellular autophagosomal machinery by RNA viruses. PLoS Biol 3:e156. doi:10.1371/journal.pbio.0030156. 15884975 PMC1084330

[8] Feng Z, Hensley L, McKnight KL, Hu F, Madden V, Ping L, Jeong SH, Walker C, Lanford RE, Lemon SM. 2013. A pathogenic picornavirus acquires an envelope by hijacking cellular membranes. Nature 496:367–371. doi:10.1038/nature12029. 23542590 PMC3631468

[9] Robinson SM, Tsueng G, Sin J, Mangale V, Rahawi S, McIntyre LL, Williams W, Kha N, Cruz C, Hancock BM, Nguyen DP, Sayen MR, Hilton BJ, Doran KS, Segall AM, Wolkowicz R, Cornell CT, Whitton JL, Gottlieb RA, Feuer R. 2014. Coxsackievirus B exits the host cell in shed microvesicles displaying autophagosomal markers. PLoS Pathog 10:e1004045. doi:10.1371/journal.ppat.1004045. 24722773 PMC3983045

[10] Chahar HS, Bao X, Casola A. 2015. Exosomes and their role in the life cycle and pathogenesis of RNA viruses. Viruses 7:3204–3225. doi:10.3390/v7062770. 26102580 PMC4488737

[11] Matsumoto J, Stewart T, Banks WA, Zhang J. 2017. The transport mechanism of extracellular vesicles at the blood-brain barrier. Curr Pharm Des 23:6206–6214. doi:10.2174/1381612823666170913164738. 28914201

[12] Wang Y-F, Yu C-K. 2014. Animal models of enterovirus 71 infection: applications and limitations. J Biomed Sci 21:31. doi:10.1186/1423-0127-21-31. 24742252 PMC4013435

[13] Ong KC, Badmanathan M, Devi S, Leong KL, Cardosa MJ, Wong KT. 2008. Pathologic characterization of a murine model of human enterovirus 71 encephalomyelitis. J Neuropathol Exp Neurol 67:532–542. doi:10.1097/NEN.0b013e31817713e7. 18520772

[14] Théry C, Witwer KW, Aikawa E, Alcaraz MJ, Anderson JD, Andriantsitohaina R, Antoniou A, Arab T, Archer F, Atkin-Smith GK, Ayre DC, Bach J-M, Bachurski D, Baharvand H, Balaj L, Baldacchino S, Bauer NN, Baxter AA, Bebawy M, Beckham C, Bedina Zavec A, Benmoussa A, Berardi AC, Bergese P, Bielska E, Blenkiron C, Bobis-Wozowicz S, Boilard E, Boireau W, Bongiovanni A, Borràs FE, Bosch S, Boulanger CM, Breakefield X, Breglio AM, Brennan MÁ, Brigstock DR, Brisson A, Broekman ML, Bromberg JF, Bryl-Górecka P, Buch S, Buck AH, Burger D, Busatto S, Buschmann D, Bussolati B, Buzás EI, Byrd JB, Camussi G, Carter DR, Caruso S, Chamley LW, Chang Y-T, Chen C, Chen S, Cheng L, Chin AR, Clayton A, Clerici SP, Cocks A, Cocucci E, Coffey RJ, Cordeiro-da-Silva A, Couch Y, Coumans FA, Coyle B, Crescitelli R, Criado MF, D'Souza-Schorey C, Das S, Datta Chaudhuri A, de Candia P, De Santana EF, De Wever O, Del Portillo HA, Demaret T, Deville S, Devitt A, Dhondt B, Di Vizio D, Dieterich LC, Dolo V, Dominguez Rubio AP, Dominici M, Dourado MR, Driedonks TA, Duarte FV, Duncan HM, . 2018. Minimal information for studies of extracellular vesicles 2018 (MISEV2018): a position statement of the International Society for Extracellular Vesicles and update of the MISEV2014 guidelines. J Extracell Vesicles 7:1535750. doi:10.1080/20013078.2018.1535750. 30637094 PMC6322352

[15] Ayloo S, Gu C. 2019. Transcytosis at the blood-brain barrier. Curr Opin Neurobiol 57:32–38. doi:10.1016/j.conb.2018.12.014. 30708291 PMC6629499

[16] Smither SJ, Lear-Rooney C, Biggins J, Pettitt J, Lever MS, Olinger GG, Jr. 2013. Comparison of the plaque assay and 50% tissue culture infectious dose assay as methods for measuring filovirus infectivity. J Virol Methods 193:565–571. doi:10.1016/j.jviromet.2013.05.015. 23748121

[17] Yue Y, Li P, Song N, Li B, Li Z, Guo Y, Zhang W, Wei MQ, Gai Z, Meng H, Wang J, Qin L. 2016. Genomic and immunologic factors associated with viral pathogenesis in a lethal EV71 infected neonatal mouse model. Mol Med Rep 13:4183–4190. doi:10.3892/mmr.2016.5080. 27035332 PMC4838153

[18] Mao L, Wu J, Shen L, Yang J, Chen J, Xu H. 2016. Enterovirus 71 transmission by exosomes establishes a productive infection in human neuroblastoma cells. Virus Genes 52:189–194. doi:10.1007/s11262-016-1292-3. 26837894

[19] Murgoci AN, Duhamel M, Raffo-Romero A, Mallah K, Aboulouard S, Lefebvre C, Kobeissy F, Fournier I, Zilkova M, Maderova D, Cizek M, Cizkova D, Salzet M. 2020. Location of neonatal microglia drives small extracellular vesicles content and biological functions in vitro. J Extracell Vesicles 9:1727637. doi:10.1080/20013078.2020.1727637. 32158520 PMC7049881

[20] Mensa E, Guescini M, Giuliani A, Bacalini MG, Ramini D, Corleone G, Ferracin M, Fulgenzi G, Graciotti L, Prattichizzo F, Sorci L, Battistelli M, Monsurro V, Bonfigli AR, Cardelli M, Recchioni R, Marcheselli F, Latini S, Maggio S, Fanelli M, Amatori S, Storci G, Ceriello A, Stocchi V, De Luca M, Magnani L, Rippo MR, Procopio AD, Sala C, Budimir I, Bassi C, Negrini M, Garagnani P, Franceschi C, Sabbatinelli J, Bonafe M, Olivieri F. 2020. Small extracellular vesicles deliver miR-21 and miR-217 as pro-senescence effectors to endothelial cells. J Extracell Vesicles 9:1725285. doi:10.1080/20013078.2020.1725285. 32158519 PMC7048230

[21] Tan Q, Shi S, Liang J, Zhang X, Cao D, Wang Z. 2020. MicroRNAs in small extracellular vesicles indicate successful embryo implantation during early pregnancy. Cells 9:645. doi:10.3390/cells9030645. PMC7140406 32155950

